# Understanding pain in modern society: insights from attitudes to pain in the Medieval Period

**DOI:** 10.3389/fpain.2023.1162569

**Published:** 2023-05-09

**Authors:** Emma G. Paley, Mark I. Johnson, Carole A. Paley

**Affiliations:** ^1^ Institute for Medieval Studies, University of Leeds, Leeds, United Kingdom; ^2^ Centre for Pain Research, School of Health, Leeds Beckett University, Leeds, United Kingdom; ^3^ Academic Unit of Palliative Care, University of Leeds, Leeds, United Kingdom

**Keywords:** pain, medieval, history, painogenic environment, social cohesion, ascetics, attitudes and behaviors

## Abstract

Historical records provide knowledge about the way people lived in the past. Our perspective is that historical analyses of the Medieval Period provide insights to inform a fuller understanding of pain in the present era. In this article, we appraise critiques of the writings of people living with pain during the mid (high) to late Medieval Period (c. 1,000–1,500 AD) to gain insights into the nature, attitudes, lived experience, and sense-making of pain. In the Medieval Period, pain was understood in terms of Galen's four humours and the Church's doctrine of pain as a “divine gift”, “punishment for sin” and/or “sacrificial offering”. Many treatments for pain were precursors of those used in modern time and society considered pain to be a “shared experience”. We argue that sharing personal stories of life is a fundamental human attribute to foster social cohesion, and that nowadays sharing personal stories about pain is difficult during biomedically-focussed time-constrained clinical consultations. Exploring pain through a medieval lens demonstrates the importance of sharing stories of living with pain that are flexible in meaning, so that people can connect with a sense of self and their social world. We advocate a role for community-centred approaches to support people in the creation and sharing of their personal pain stories. Contributions from non-biomedical disciplines, such as history and the arts, can inform a fuller understanding of pain and its prevention and management.

## Introduction

Exploring pain through a historical lens offers insights into human understanding, thought and expression, and can provide perceptions of relationships between human biology and sociocultural conventions. The Medieval Period is one of the three traditional divisions of Western history (antiquity, medieval, modern) and a time of great religious, cultural and social development in Europe, paving the way for new scientific thinking. In this perspectives article we examine the meanings attributed to pain and the attitudes and responses to pain during the mid (high) to late Medieval Period. We will discuss the possible mindsets of medieval people experiencing pain and discuss how this may inform a fuller understanding of pain in modern society.

## Pain in the Medieval Period

The Medieval Period (Middle Ages) began with the fall of the Western Roman Empire (c. 476 AD) and transitioned into the Renaissance period (c. 1,500 AD). During this time, approximately 90% of the population were peasants (villeins) working the land and living in small communities under the control of overlords. Much of Europe had become Christian and the first universities were established.

The Medieval Period is divided into: the Early Middle Ages (c. 425–1,000 AD); the High Middle Ages (c. 1,000–1,300 AD); and the Late Middle Ages (c. 1,300–1,500 AD). The bubonic plague (Black Death) occurred during the Late Middle Ages and was associated with mortality of over 20 million people, 30%–50% of the continent's population. A common view in society was that the plague was God's punishment for sin, although some believed that it was a result of an astrological event or an earthquake which released poisonous vapours (1).

### Knowledge and attitudes

**Figure 1:**
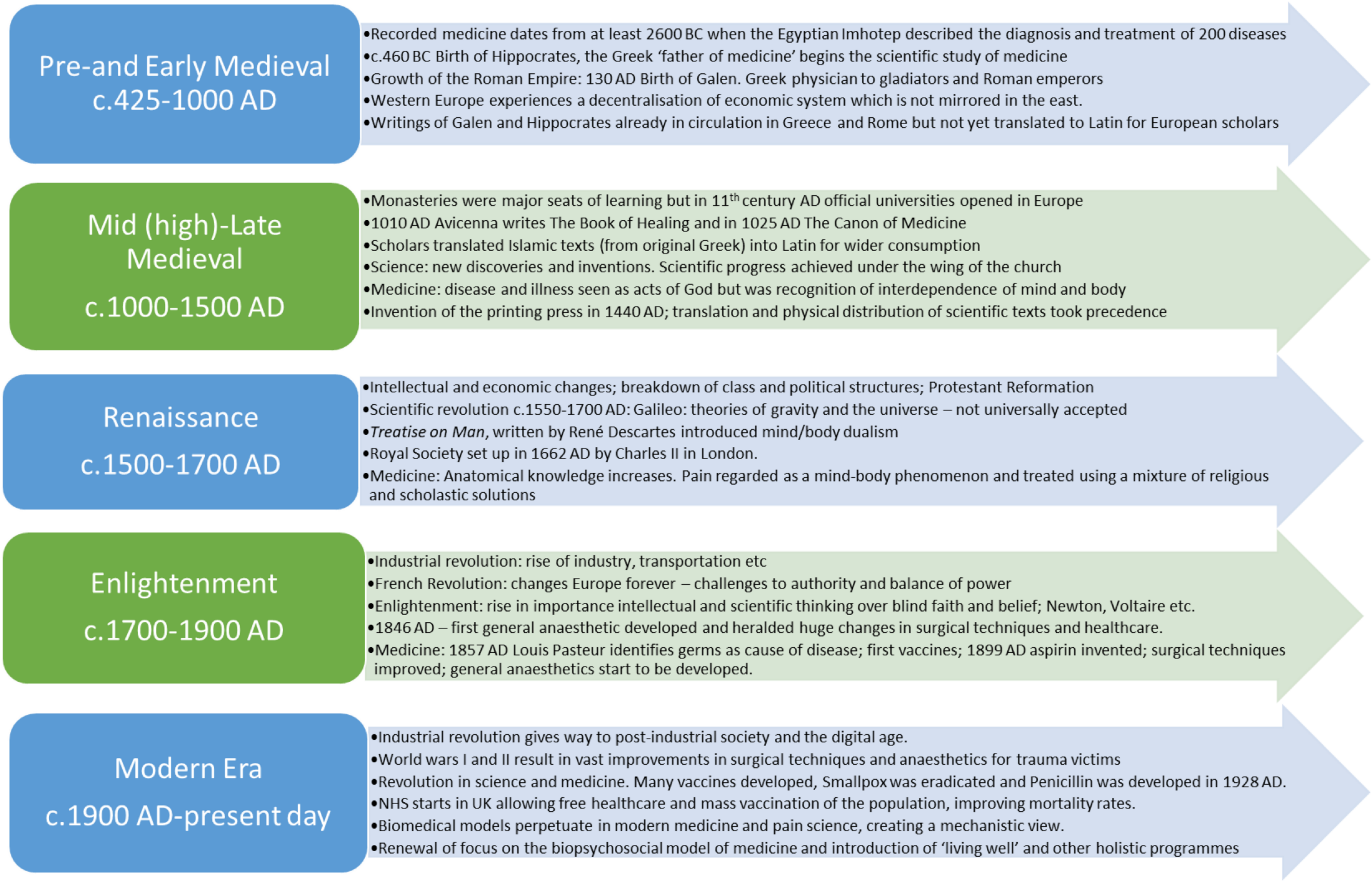
A contextual history of scientific and medical understanding.

The book *A History of Pain* by Rey provides a synopsis of institutional and scientific conditions in which theories and knowledge about pain were made (2). Before the Medieval Period Hippocrates (c. 460 − c. 377 BC) argued that diseases were caused naturally, and not because of superstition and gods. The Greek physician and philosopher Galen (c. 129–216 AD) described pain as a “rupture of continuity” or a “change in temperament” caused by an imbalance of the four humours: blood, phlegm, yellow and black bile. Galen believed the mind/soul and body were intimately interconnected and therefore involved in the experience of pain (3). The *Treatise on Man,* published by René Descartes in the 17th century, differentiated the body and the mind (or soul). This catalysed a biomechanistic model of pain which paved the way for modern medicine but may have marginalised the significance of the mind (4). The Medieval Period spans Galenism with its focus on anatomy and the four humours, and the early modern period with Descartes’ mechanistic model of pain (Figure 1).

Throughout history pain has been considered “a passion of the soul”. Acute and chronic are relatively recent additions to the pain lexicon and generally physicians only became interested in chronic pain without obvious pathology in the 1900s, with people complaining of long-term pain often regarded as deluded or malingerers. Thus, historical texts discuss long-term illness and/or pain but do not describe pain in terms of “acute” or “chronic”.

During the Medieval Period, knowledge and attitudes towards pain and suffering arise from biographical sources (*vitae*) of historical figures, and occasionally some autobiographical details, although these were rare due to high levels of illiteracy. Most knowledge originates from religious establishments. Convents were one of the few places where women could receive an education, and nuns wrote, translated, and illuminated manuscripts. It is largely from these sources, which were heavily influenced by the Christian beliefs and culture of the time, that an understanding of pain in the Medieval Period is informed. However, early scribes probably exaggerated, diminished, added, or removed events from the accounts of the lives of individuals, so caution is needed in interpretation.

In the Medieval Period, pain was frequently written about with a scholastic or devotional theme, or both, as seen in letters written by the Benedictine Abbess Hildegard von Bingen (1,098–1,179 AD), a medieval visionary and mystic to those who sought her medical advice (5). These letters revealed attitudes towards pain and illness during the Medieval Period; there were no straightforward causal relationships and they involved both the body and the mind. Even when trained physicians were available from around the 12th century, most people were unable to pay and therefore sought treatments from untrained healers and through religious means.

Treatment for pain was largely reliant on traditional folklore, superstition and herbal tinctures (6). Physicians used astrological charts to aid diagnosis and treatment. In the late 11th Century, new ideas were imported into Europe, probably as a result of the first Crusade. Islamic scientific and medical texts (originally from Greece) were translated into Latin so that they could be read by western European scholars. At this time, Avicenna (*Ibn Sina*), a Persian polymath (980–1,037 AD) followed Galenic thinking and published “*The Canon of Medicine”* in 1,025 AD (7) which set the standard for medicine in medieval Europe and the Islamic world into the 18th century. Within “*The Canon”*, Avicenna challenged some aspects of Galen's work and argued that pain was not always an “interruption of continuity”, and that bodily adaptation could occur in the presence of pain (8). Although medieval texts did not distinguish between chronic and acute pain as such, some writers referred to long-term painful illnesses.

In the 12th century, the Andalucian polymath and physician and philosopher Averroes (*Ibn Rushd*) wrote *The Book of the Principles of Medicine (The Kulliyat)* which recognised observation rather than mere speculation in the diagnostic process (9) suggesting early practise of evidence-based medicine (10). Guy de Chauliac defined pain in his *Grande Chirurgie* (1,363 AD): “*Pain, according to Avicenna, is a feeling of contradictory qualities. But along with these contradictory humors which might inflict pain, according to Galen, there may be alterations which break or cut, stretch or abrade: pain is therefore the result either of personally generated contrary qualities, or interruptions in continuity caused by accidents*”. [Cited in (2)].

### Lived experience

Rey claims that there are few accounts of how individuals experienced pain and suffering until the shift in religious preoccupations in the 12th century (2). Figurative scenes of endurance of agony, pain, and suffering of saints, as depicted on stained glass windows, offered clues about the societal relationship with pain. Medieval society was ordered by powerful men of church authorities or feudal lords warring with one another. Rey speculates that during this era there would be little time to ruminate on pain experience. Christianity positioned itself as a religion of salvation and healing through faith and prayer. Rey argues that this social milieu would provide little space for “intimate attention to the body” and encouraged a stoic indifference to pain.

Rey's views of stoicism and indifference to pain are contested by Cohen who devotes an entire chapter of the book *The Modulated Scream: Pain in late Medieval Culture*, to impassibility; mainly of the martyrs but also of those undergoing torture …. “*They did suffer; they did not possess miraculous impassibility”* (11). Cohen argues that written accounts of the pain of others was speculative and that any apparent indifference to pain must have been an ability to withstand it. It is unlikely that medieval people had the ability to be indifferent to pain and would utilise various strategies and narratives to cope with it. Religious and scholastic attitudes towards pain and disease were so intertwined during this period that people would have tried various strategies for relief. “Saintly stoicism” was probably confined to a few individuals, such as the mystics and pious religious figures.

Cohen draws attention to a difference between people experiencing pain in the late Medieval Period (c. 1,300–1,500 AD) and those of modern time; referring to the social milieu of living with pain in the modern era as “*utter isolation and solitude of the sufferer*” (12). Cohen states “*The modern sufferer is trapped inside her pain, unable to share or express it. In contrast, in the later Middle Ages pain was definitely a social sensation … pain was shared, discussed and transmitted through speech, art and patterns of behaviour*” (12). Cohen argues that sharing pain with others fostered social cohesion and solidarity amongst similar social groups, such as the small, impoverished village communities. The Renaissance and the scientific revolution (c. 1,550 AD) grounded an understanding of pain in bodily pathophysiological disruption, locating pain and its treatment within tissue. People not responding to biomedical treatments were left isolated, disorientated, and helpless by an indifferent and uncomprehending medical paradigm; over time these sentiments spread in the wider social world (13). In the modern era, people continue to share pain experience with family and friends, and within cultural, religious, and societal groups which mirrors the medieval experience of “shared suffering”; however, constraints on resources and the need to quantify pain means that sharing pain experience remains marginalised in health service delivery.

### Sense-Making

In the mid to late Medieval Period the pain of Christ was an important part of sense-making. medieval people interpreted the church's premise that pain was a “divine gift” or “sacrificial offering” to get closer to God or as a means of punishment and redemption in various, often contradictory ways. The mystic Beatrice of Nazareth (c. 1,200–1,268 AD) wrote that her many illnesses were a blessing and her pain was a way of being tested and to get closer to God (14). The visionary Margery Kempe (c. 1,373–1,439 AD) thought her painful illnesses were a punishment for being an imperfect human rather than for any specific sin (15). Kempe rationalises the unpredictability of her pain by attributing its origin to God, although she was not affiliated with any religious order.

Medieval *vitae* of the ascetics describe how they practiced severe self-denial and self-infliction of pain either as a form of self-punishment or to mimic the suffering of Christ, possibly through altering their conscious state, in order to be morally acceptable before the divine (16, 17). The mystic and Augustinian Marie d'Oignies (c. 1,177–1,213 AD) self-inflicted pain as a means of punishment and to develop her spiritualty (18), and she overcame this pain claiming that she “had been so inflamed by the overwhelming fire of love” (of God) (18). medieval mystics and others, such as religious martyrs appear to have been able to divert their attention away from pain, possibly by thought suppression and self-hypnosis (17), similar to that observed in modern times, e.g., sport ultra-endurance athletes (19) or extreme sports protagonists (20). In both medieval and modern times context would determine whether such behaviours of mystics and ascetics were perceived as a psychiatric disorder, a feat of “strong will” or the intervention of a supernatural force (21).

### Alleviating pain

During the mid to late Medieval Period the first universities in Europe were established. Trained physicians mostly tended to those who could afford to pay. The Universities were affiliated with the Church and scholars were expected to take minor orders, thus forming a complex theology/medicine relationship in medieval Europe (3). A debate about the tension between the Christian “suffering self” and the desire to relieve pain by any means during the Medieval Period remains unresolved (3, 12).

The prevailing Christian view, that pain was a punishment for sin or a divine intervention worthy of reward in the afterlife, fostered an attitude that pain was something to be endured. Nevertheless, evidence suggests that medieval people *suffered* pain and wanted relief from it. Importantly, painful illness which prevented people from working the land had financial consequences because rents to overlords and tithes to the church could not be paid. This provided a strong incentive to find relief from pain and also placed reliance on small village communities to support the ill and infirm (22). It has widely been thought that life expectancy was only 30–35 years during the Medieval Period, but this has now been shown to be incorrect and skewed due to high infant mortality. Those living to the age of 25 had a good chance of surviving until they were 50 and possibly much longer (23, 24). Therefore, they would have a greater likelihood of experiencing pain and illness, and possibly for a prolonged period of time.

Spiritual relief of pain was often sought by an array of religious activities including prayer, pilgrimages and seeking miracles all of which continue into modern times (25). John of Mirfield (1,362–1,407 AD), amongst others, understood the desire for pain relief and that pain in itself could result in further illness or death (26). Nevertheless, in some circumstances pain should be borne without relief as it was believed that interventions to alleviate pain would interfere with natural processes e.g., by causing contractions to stop during painful childbirth (27).

Medieval healers often used painful antiquated treatments such as bloodletting and other types of purging to rid the body of noxious substances, balance the humours and to ‘drain away sins’. In 1,363 AD, Guy de Chauliac's *Grande Chirurgie* described principles for treatment based on “opposites” to counteract disorders including pain, e.g., humidity for dryness, heat to “ward off cold” (28). Guy de Chauliac advocated evacuation or purges and remedies to inflame or suppurate using fats and oils, mixed with bread and eggs, and applied as plasters to defuse heat. He also used ligatures to render painful body parts insensate and to prevent bleeding. The acceptance of painful procedures to cure pain continues to modern times, e.g., surgery, emetics, laxatives, and the draining of bodily fluids such as cysts.

Methods of soothing pain during the Medieval Period included sparing use of plants such as hemlock or opium (29). The earliest version of the Old English Herbal is the Cotton MS Vitellius C III, written in the early 11th century, describing plants and their uses. The Antidotarium Nicolai, written between 1,160–1,220 AD, distinguished between antidotes for pain and those treating illness and was written as a guide to the ingredients required for popular remedies (30). Examples included sponges infused with narcotic substances applied to the skin prior to incision or inhaled as gases through the nose. These procedures echo modern-day analgesic practices such as the use of morphine patches or inhaled Entonox. Hildegard von Bingen (1,098–1,179 AD), Benedictine abbess of the Rhineland in Germany, was a visionary, mystic and healer, that produced remedies for a multitude of ailments using some substances still in use today (31). Some remedies contained dangerous substances such as mandragora root (mandrake), nightshade, and henbane that were administered in small quantities. Some became the precursors of modern-day analgesic agents, for example, opium and willow bark (containing salicylic acid).

It was believed that people undergoing surgery in the Medieval Period received no relief of pain because it had been thought that there were no effective anaesthetics in England until approximately 150 years ago. However, the use of anaesthetics pre-dates Roman times in southern Europe (c. 800 AD) (32, 33). Late medieval English texts (c. 12th−15th century) discovered towards the end of the 20th century contained a recipe for an anaesthetic concoction called Dwale; based on bile, lettuce, vinegar, and bryony root, hemlock, opium, and henbane. Some ingredients were highly dangerous and yet the Dwale recipe was administered by ordinary people (34) and appeared in household recipe books (35). Bryony was sometimes used as a substitute for mandrake (*Mandragora officinarum*). Mandrake could cause hallucinations and was therefore associated with magic powers and might have been responsible for out of body experiences occurring in witchcraft, although this has not been widely confirmed (36). Jeanne d'Arc (d. 1,431 AD) was accused of carrying mandrake at her trial (37).

## Discussion

The complex interchange between medical and Christian beliefs and the debate about the relative influence of medical thought on scholastic theology made the Medieval Period an interesting time in the history of pain. We have used in-depth analyses of the writings of other scholars to gain insights of the mindset of people living in the Medieval Period as summarised in Table 1.

**Table 1:** Comparison of attributes of pain in medieval and modern periods.

	Medieval Period	Modern Period (including present-day)
Dates of period	• 5th Century A.D. (fall of western Roman Empire) to c. 1,500 A.D. (start of Renaissance period) • Mid (high) to late Medieval Period started c. 1,000 A.D.	• Early modern period began c. 1,500 and late modern period began c. mid-18th century A.D. • Contemporary history began 1,945 A.D. following the second world war
Major events	• Seats of learning; monasteries and the first universities • 1,025 A.D.—Canon of Medicine (Avicenna) set standard for medicine in medieval Europe and the Islamic world • Late 12th century *The Book of the Principles of Medicine* (Averroes) • 1,160–1,220 A.D.—The *Antidotarium Nicolai* guide to the ingredients for remedies that distinguished antidotes for pain and illness • First crusades—Islamic medical documents translated into Latin for European Scholars • Bubonic plague (c. 1,346 to 1,353 A.D.)	• c. 1,550–1,700 A.D.—start of scientific revolution • Circa 1,600 A.D.—Descartes–Cartesian dualism fostered mechanistic biomedical model of healthy body and denied significance of mind • 1,846 A.D.—advent of anaesthetics • 1,950 A.D. onwards—emergence of influential pain specialists/scientists e.g., Bonica, Melzack and Wall, Woolf etc.
Pain experts/influencers	• The church • Local healers • Medical scholars (later Medieval Period)	• Medical practitioners/specialists • Registered and unregistered therapists/healers • Pain specialists • Social media
Phenomenology of pain	• “Social sensation” • Shared experience • Coherent with shared life demands and expectations of community	• “Individual sensation” • Private experience • Coherent with damaged body needing medical attention creating expectation of diagnosis and cure
Phenomenology of suffering	• Social and public suffering within a cohesive family and community unit	• Suffering in isolation and solitude perhaps reflecting some fragmentation of family and community units
Meaning of pain	• Multiple meanings • Humoural imbalance • Result of treatment or process of healing • Blessing from God • Devine punishment for sin, a penitence, retribution, punishment, or martyrdom	• Single meaning (i.e. biomedically dominant) • Tissue damage or dysfunctional physiology • Present-day recognition of biopsychosocial influences
Ontology	• Holistic, part of a whole person, including the personality • Carried within the soul	• Materialistic, body parts and biomedical constituents • Produced by the brain
Explanatory model	• No straightforward causal explanation • Humoural imbalance involving body, mind and/or soul • God / Metaphysical processes • Unclear whether medical or scholastic attitudes were separate or intertwined	• Symptom of pathology • Neuro-mechanistic processes with biopsychosocial influences • Dysfunctional somatosensory system
Expression of pain	• Verbalisation, behaviour and artform rooted in diverse narratives	• Verbalisation and behaviour predominantly rooted in biomedical narrative
Societal attitude	• Tension between Christian “suffering self” and desire to relieve pain • Pain from illness required alleviation • Pain from surgery, or childbirth should not be treated • Endure pain because ‘from God’ • Spiritual relief by prayer, pilgrimages, miracles and religious power	• Relief of pain is a human right • Expectation of a cure • Biomedical, and more recently psychosocial, approaches to ‘fix’ body and mind • Pain as a technical problem • Treatment ‘failure’ if individual remains in pain
Individual hopes, beliefs and expectations	• Hope for relief and possibility of cure • Fear pain could result in further illness or death • Treatment failure—‘God's Will’ • Behaviour—short period of therapy shopping • Continue to work to survive • Low expectation of complete relief and return to ‘normal’/’optimal’ health	• Expectation of relief and of cure • Fear pain could signal sinister disease • Treatment failure—multiple explanations, incorrect treatment, poor medical practice, complex medical condition • Behaviour—prolonged therapy shopping … symptom relief whilst searching for diagnosis and cure • Absence from work—illness benefits • High expectation of complete relief and return to ‘normal’/’optimal’ health
Asceticism	• Often by religious leaders, mystics, martyrs experiencing torture to be morally acceptable before the divine	• Often by sportspeople, military to be stoic, competitive or exhibitionistic
Forces of power	• The Church • Social power	• Biomedicine/health care • Medical power
Pain practitioners	• Local trained and untrained healers and ‘wise women’ using folklore • Mystics and religious orders (in case pain resulted from sin) • Physicians (medical scholars) available c. 12th century A.D., often aided by astrological charts—but too expensive for most people	• Physicians, health care practitioners, multidisciplinary teams, using biomedical diagnosis • CAM practitioners • Religious orders • Untrained and unregistered healers
Modes of treatment	• Access to medical care very limited • Traditional folklore, herbal tinctures, external concoctions, plants (hemlock, opium, willow bark), salves and plasters • Purging to rid the body of noxious substances or drain away sins • Balance humours	• Access to medical care widespread • Treatment targeting biomedical constituents such as anaesthetics, analgesics, pain adjuvants and surgery • Biopsychosocial approaches and health promoting and lifestyle adjustments
Pain writings	• Scholastic and devotional theme • Often only available to educated few	• Applied biomedical/psychosocial theme • Knowledge generated by pain specialists/scientists and available to society

In the Medieval Period, pain was a multifaceted shared social experience with several meanings, and people sought to alleviate pain using physical, spiritual, and social interventions. Sharing pain promotes social bonding, cooperative behaviour, camaraderie, and well-being (38–40). Nowadays, people report feelings of being “trapped” inside a painful “damaged” body likened to incarceration in prison and resulting in self-imposed isolation (41). Self-isolation is an evolutionary adaptation that aids survival following injury, and people will have self-isolated in the Medieval Period, although this seems to have been heightened in modern times. The rise in individualism and the inability to adequately share pain in health care settings appear to be contributing factors (42, 43). Conversely, technological developments have enabled sharing of pain via the world wide web and social media, enabling global reach way beyond the confines of local groups and communities. Sharing pain in this way may have a profound impact on pain experience, and research on the topic is in its infancy. Likewise, a bewildering multitude of choices and opinions are available nowadays for people experiencing pain. This may provide greater opportunities for recovery but may also increase the sense of isolation and hopelessness when treatments fail. We advocate a need to allow society, including health care systems, to provide opportunities for modern-day people to share pain, through for example, telling stories of pain experience using various vocabularies. Contemporary approaches to assist people on a healing journey are delivered using clinical and non-clinical personnel in settings that are “non-threatening” including the arts and visual imagery (44–46).

Medieval explanations of pain residing “within the soul” have parallels with contemporary concepts of “inner-self”, “embodied pain” and “body-mind theory” (47). Medieval humoural theory is a rudimentary framework for contemporary concepts associated with balance of the body and mind and the connection to the natural and built environments (48, 49). The shift from Galen's holistic view of pain resulting from humoural imbalance to a neuro-mechanistic model of pain has provided great advances in the understanding of nociception, sensitisation, bioplasticity and neuroimmune function. Neilson argues that the neuro-mechanistic view of pain is an “illusion of great scientific progress” because the vast accumulation of physiological knowledge conceals a model that does not explain the *subjective* experience of pain i.e., the hard problem of consciousness (50). A consequence of conflating nociception (neurophysiology) and pain (51) has been to decontextualise physiological processes from the lived experience (42) resulting in neglect of the socio-ecological factors that shape a person's lifeworld and contribute to painogenicity (13, 52).

Contemporary models describe pain is an emergent phenomenon of brain activity rather than an identifiable “thing” (51, 53–56). Calls to reflect social and phenomenological aspects of pain in scientific definitions (57, 58), consistent with the shared social experience of pain in the Medieval Period, are growing. Bourke argues that pain should be considered a “… *type of an event … one of those recurring occurrences that we regularly experience and witness that participates in the constitution of our sense of self and other*” (59) p. 5. Our appraisal suggests that pain would have been considered more like a “type of event” than a “thing” in the Medieval Period.

Under the power of the Church's narrative, failure to relieve pain in the Medieval Period was probably interpreted as “God's will”; an attitude which remains to this day in some cultures and communities. We speculate that this may have fostered an acceptance of the need to endure pain without relief. The biomedical paradigm which has driven advances and refinements of the medieval pharmacopeia has raised societal hope and expectations of relief (and cure). Advances in biomedicine have produced a wealth of beneficial pain treatments, yet unremitting pain and suffering remains a major challenge of the modern period. Forces controlling societal narratives about pain (e.g., the Church or biomedicine) have, to some extent, disenfranchised people. We argue greater focus on investigation of “upstream” factors, such as societal narrative, that may be creating painogenic environments, as this is likely to assist prevention of pain and its persistence. We also advocate a need to empower people to take control of their own pain story (60), with a role for community-centred biopsychosocial approaches to assist recovery and to live well with pain (61). Contemporary approaches to de-marginalise people in pain include a recognition that the arts (45, 46), including the use of imagery, aid understanding of the lived experience of pain (44) and give meaning to life itself: “*If health is about adaptation, understanding, and acceptance, then the arts may be more potent than anything that medicine has to offer.”* (46)

## Conclusion

The medieval perspective of pain provides insights for a fuller understanding of the socio-ecological conditions contributing to a painogenic milieu, offering insights to upstream strategies to prevent pain. Severe physical hardship was common for many people during the Medieval Period (i.e., in Europe) and pain was probably common, with chance of relief low. Improvements in living standards and in pain treatment have not resolved the burden of unremitting pain in society. In some ways, the mindset of medieval people toward pain parallels people in the modern era; people seek relief under the constraints of affordability, availability and acceptability and guided by therapeutic, community and theological beliefs. Personal life-worlds about pain are constructed within the social narratives of the time, and many medieval narratives survive to the present day in refined forms. Pain as a shared experience is a longstanding characteristic of human communities. This supports the need for flexibility in modern-day explanations of pain that are acceptable to individuals and communities, so that they can connect with a sense of self and the social world (62). To do this, we advocate exploration of pain and its management via an eclectic mix of subject disciplines, including history, the arts and storytelling, which would help patients validate their pain and allow them to express psychological and spiritual aspects of their experiences (63).

## Accessing research materials

Underlying materials related to our paper can be accessed by contacting Professor Mark I. Johnson.

## Data Availability

The original contributions presented in the study are included in the article, further inquiries can be directed to the corresponding author.
